# Performance of *in silico* prediction tools for the classification of rare *BRCA1/2* missense variants in clinical diagnostics

**DOI:** 10.1186/s12920-018-0353-y

**Published:** 2018-03-27

**Authors:** Corinna Ernst, Eric Hahnen, Christoph Engel, Michael Nothnagel, Jonas Weber, Rita K. Schmutzler, Jan Hauke

**Affiliations:** 1 Center for Familial Breast and Ovarian Cancer, Center for Integated Oncology (CIO), Medical Faculty, University Hospital Cologne, Kerpener Straße 34, Cologne, 50931 Germany; 2 Institute of Medical Informatics, Statistics and Epidemiology (IMISE), Leipzig, Germany; 3 Cologne Center for Genomics, University of Cologne, Cologne, Germany

**Keywords:** BRCA, Classification, Missense variant, Prediction tools, Variant of uncertain significance

## Abstract

**Background:**

The use of next-generation sequencing approaches in clinical diagnostics has led to a tremendous increase in data and a vast number of variants of uncertain significance that require interpretation. Therefore, prediction of the effects of missense mutations using *in silico* tools has become a frequently used approach. Aim of this study was to assess the reliability of *in silico* prediction as a basis for clinical decision making in the context of hereditary breast and/or ovarian cancer.

**Methods:**

We tested the performance of four prediction tools (Align-GVGD, SIFT, PolyPhen-2, MutationTaster2) using a set of 236 *BRCA1/2* missense variants that had previously been classified by expert committees. However, a major pitfall in the creation of a reliable evaluation set for our purpose is the generally accepted classification of *BRCA1/2* missense variants using the multifactorial likelihood model, which is partially based on Align-GVGD results. To overcome this drawback we identified 161 variants whose classification is independent of any previous *in silico* prediction. In addition to the performance as stand-alone tools we examined the sensitivity, specificity, accuracy and Matthews correlation coefficient (MCC) of combined approaches.

**Results:**

PolyPhen-2 achieved the lowest sensitivity (0.67), specificity (0.67), accuracy (0.67) and MCC (0.39). Align-GVGD achieved the highest values of specificity (0.92), accuracy (0.92) and MCC (0.73), but was outperformed regarding its sensitivity (0.90) by SIFT (1.00) and MutationTaster2 (1.00). All tools suffered from poor specificities, resulting in an unacceptable proportion of false positive results in a clinical setting. This shortcoming could not be bypassed by combination of these tools. In the best case scenario, 138 families would be affected by the misclassification of neutral variants within the cohort of patients of the German Consortium for Hereditary Breast and Ovarian Cancer.

**Conclusion:**

We show that due to low specificities state-of-the-art *in silico* prediction tools are not suitable to predict pathogenicity of variants of uncertain significance in *BRCA1/2*. Thus, clinical consequences should never be based solely on *in silico* forecasts. However, our data suggests that SIFT and MutationTaster2 could be suitable to predict benignity, as both tools did not result in false negative predictions in our analysis.

**Electronic supplementary material:**

The online version of this article (10.1186/s12920-018-0353-y) contains supplementary material, which is available to authorized users.

## Background

The classification of variants of uncertain significance (VUS) is a major challenge for centers performing genetic testing, e.g., in families at risk for breast or ovarian cancer. The German Consortium for Hereditary Breast and Ovarian Cancer (GC-HBOC) is a multicenter consortium of interdisciplinary university centers specialized in providing counseling, genetic testing and healthcare for familial breast and ovarian cancer. To establish and consolidate patient-centered care and research for HBOC in Germany, the consortium runs a central patient registry and is involved in defining guidelines for genetic testing, treatment and variant classification. VUS are often extremely rare variants, for instance, analysis of more than 29,316 families within the framework of GC-HBOC (as of September 2016) revealed that 64.4% of the missense VUS identified in the *BRCA1/2* genes are private. Nevertheless, classification of genetic aberrations is highly relevant for clinical decision making. For individuals at risk for breast and/or ovarian cancer, the option to undergo prophylactic surgery is limited to carriers of pathogenic mutations in relevant risk genes. In addition, for patients affected by breast and/or ovarian cancer, knowledge about their *BRCA1/2* mutation status is important because it determines the therapeutic response [1] and choice of medication (e.g., PARP inhibitors [2]). To circumvent the problem of missing information on rare genetic variants and the requirement for their interpretation, the automatized prediction of effects of missense mutations has become a frequently used approach in clinical diagnostics.

Existing *in silico* approaches for the classification of missense mutations mainly rely on the assumption that disease-associated missense mutations are (1) characterized by a large difference between the biochemical properties of substituted amino acids (AAs) and (2) located at highly conserved genomic regions across species. Based on these criteria, the available tools can be roughly divided into the following subcategories: sequence-based, if the method solely relies on assumption (1); structure-based, if the method solely relies on assumption (2); and sequence and structure-based in cases where both criteria are considered.

In the present study, we focused on the four prediction tools embedded in the commercial Alamut™Visual software v2.8 (Interactive Biosoftware, Rouen, France), which is widely used in medical genetics [3–5], namely, Align-GVGD [6, 7], SIFT [8], MutationTaster2 [9] and PolyPhen-2 [10].

Align-GVGD takes multiple sequence alignments (MSAs) as input and computes a biochemical distance score (extension of the pairwise Grantham difference, GD) as well as a conservation score (Grantham variation, GV) on each alignment column comprising a substitution. Based on the observed values of GD and GV substitutions are classified in seven classes *C*∈{0,15,25,35,45,55,65} from least likely to interfere with function to most likely to interfere with function.

As a purely sequence-based prediction tool, SIFT classifies non-synonymous single nucleotide polymorphisms (nsSNPs) on the basis of the evolutionary conservation of amino acids within protein families. At each position of an input MSA, a scaled probability for each AA substitution to occur (SIFT score) is computed. A missense variant is predicted to have a damaging effect on protein function, when the SIFT score of the substituted AA is below a threshold of 0.05.

MutationTaster2 uses regulatory features, degree of evolutionary conservation and splice site predictions as the input for a naïve Bayes classifier, which categorizes variants into either disease causing or polymorphism. Additionally, mutations that are found to be homozygous more than four times in the 1000 Genomes Project or the HapMap databases are automatically classified as a polymorphism, whereas variants marked as pathogenic in ClinVar are classified as disease causing by default.

PolyPhen-2 (Polymorphism Phenotyping v2) uses eight sequence-based and three structural features as the input for a naïve Bayes classifier, the latter being considered only in cases where a 3D structure is known for the protein of interest. The classifier can be chosen to be trained on one of two training data sets, namely HumDiv and HumVar [10].

For the user’s convenience, Alamut™Visual calls Align-GVGD, SIFT and MutationTaster2 directly with pre-defined parameters and provides a pre-filled web interface for PolyPhen-2.

In recent years, several studies have been published on the performance and reliability of existing approaches for *in silico* prediction of the functional impact of non-synonymous variants [4, 5, 11–16]. In summary, these studies revealed a diverse picture of the performance of these applications. The study by Luxembourg et al. [13] reported an increased number of misclassifications in cases where mutations were localized in the *α*-helix of a corresponding protein. Rodrigues et al. [15] found that genomic regions of strong conservation as well as hypervariability may negatively affect prediction results. Grimm et al. [11] noted that the evaluation of several tools suffered from overfitting, as variants used to train the methods also appeared in the evaluation set. A recent review by Tang and Thomas [16] on existing prediction approaches underscores the general lack of accurate benchmark data sets for the reliable evaluation of state-of-the-art approaches. Due to the specific weaknesses of each prediction tool, a common strategy is to combine the results of various approaches, i.e., assuming a disease-causing mutation when at least half of several approaches classify a variant as damaging. However, Leong et al. [13] found that such a strategy might even decrease reliability, as they demonstrated for a set of 113 nsSNPs in the human *SCN5A* gene. Hence, the present work aimed to investigate the requirements for a performance increase by the combination of several prediction tools using a data set of well characterized *BRCA1/2* variants. In particular, we studied how such combinations influence the sensitivity, specificity, accuracy and Matthews correlation coefficient (MCC) compared to stand-alone tools.

## Methods

### Curation of missense variant data sets

Different guidelines for the classification of sequence variants exist [17–19]. For the classification of missense variants in *BRCA1/2* the multifactorial probability model [20, 21] is widely accepted; classification of variants according to the 5-tier system suggested by Plon et al. [22] is the standard in most diagnostics labs worldwide. This model also serves as the basis for the *BRCA1/2* Gene Variant Classification Criteria proposed by the Evidence-based Network for the Interpretation of Germline Mutant Alleles (ENIGMA) [23, 24]. In a nutshell, *BRCA1/2* variants are assigned to either class 1 (neutral), 2 (likely neutral), 3 (uncertain), 4 (likely pathogenic), or 5 (pathogenic) based on a posterior probability of pathogenicity (Posterior P). Posterior P is calculated from the prior probability (Prior P) and a product of likelihood ratios (Product of LRs) derived from the multifactorial (combined) likelihood model initially suggested by Goldgar and co-workers [20]. Likelihood ratios are determined on the basis of segregation analysis, co-occurences with known deleterious variants, family histories and pathology profiles in a corresponding cohort, hence, these ratios are independent of any missense prediction. In contrast, Prior P values arise from *in silico* splice site predictions and missense analysis whose results are directly assigned to corresponding probability values. Align-GVGD is the commonly used missense prediction tool for the purpose of *BRCA1/2* variant classification [21, 25]. Consequently, to evaluate the performance of Align-GVGD and the other tools under consideration, we identified variants (1) that were definitely classified as (likely) benign, i.e., assigned to classes 1 or 2, or (likely) pathogenic, i.e., assigned to classes 4 or 5, due to comprehensible criteria and (2) whose classifications were independent of Prior P values. In doing so, an initial set of 236 nsSNPs was selected from the GC-HBOC database, the BRCA gene Ex-UV database (http://hci-exlovd.hci.utah.edu) and the literature [21, 26–28]. Variants were chosen because of their classification into classes 1, 2, 4, or 5. In concordance with the ENIGMA *BRCA1/2* Gene Variant Classification Criteria [24], selected nsSNPs had to have an allele frequency (AF)<0.01, as variants with an AF≥0.01 belong to class 1 (benign) by default. In addition, we excluded known spliceogenic variants. Allele frequencies were extracted separately for the cohorts of African, East Asian, South Asian, European (Finnish), European (non-Finnish) and Latino ancestry from the ExAC (Exome Aggregation Consortium) Browser [29], excluding TCGA (The Cancer Genome Atlas) data. For variants not listed in ExAC, the AF was set to zero. We termed the initial set of 236 nsSNPs the Classified Variant Set. Assigned variants are listed in Additional file 1: Table S1.

The dependence of Prior P and the Product of LRs on Posterior P is given by the following equations:

$$\text{Posterior Odds} = \text{Product of LRs}\ \frac{\text{Prior P}}{1-\text{Prior P}} $$

$$\text{Posterior P} = \frac{\text{Posterior Odds}}{\text{Posterior Odds} +1} $$

Consequently, given Prior P, the Product of LRs required to achieve a certain Posterior P can be determined by the numerical solution of

1 $$  \text{Posterior P} = \frac{\text{Product of LRs}\ \frac{\text{Prior P}}{1-\text{Prior P}}}{\text{Product of LRs}\ \frac{\text{Prior P}}{1-\text{Prior P}}+1}  $$

**Fig. 1:**
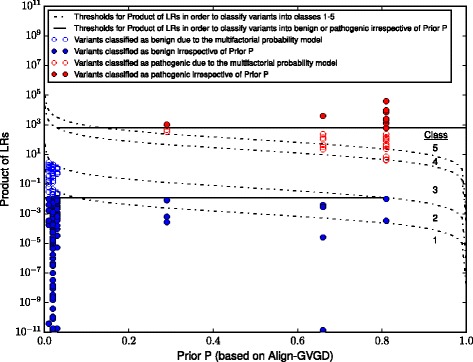
Multifactorial probability model and determination of Product of LRs thresholds for classification irrespective of Align-GVGD. Visualization of the multifactorial probability model for classification of VUS in *BRCA1/2* based on the Posterior P thresholds proposed by Plon et al. [22]. Variants with Products of LRs below or above the corresponding thresholds (indicated with filled circles) were classified independent of the prior probabilities Prior P (based on Align-GVGD predictions) and included in the Evaluation Variant Set and the Classified Variant Set. Variants depicted by unfilled circles were included in the Classified Variant Set exclusively. Classification of these variants was not independent of prior probabilities

Figure 1 shows the thresholds of the Product of LRs for the classification into one of the five pathogenicity classes in dependence to Prior P. Lindor et al. [21] proposed the assignment of a Prior P of 0.81 for Align-GVGD class 65 and 0.03 for Align-GVGD class 0. By setting Prior P in Eq. (1) to these values, we determined the ranges of values of the Product of LRs allowing for the classification into a pathogenicity class ≠3 and irrespective of Align-GVGD results. Please refer to Fig. 1 for a visualization of our approach. The Posterior P thresholds proposed by Plon et al. [22] were used, which result in a Product of LRs < 0.01 for classification as (likely) neutral and a Product of LRs > 614.33 for classification as (likely) pathogenic. We identified a total of 151 variants from our Classified Variant Set for which the Products of LRs were below or above these thresholds. Together with 10 variants that were classified by the GC-HBOC expert panel on the basis of functional analysis or additional published evidence, these variants represent our Evaluation Variant Set. The Evaluation Variant Set consists of 161 variants, namely, 89 *BRCA1* variants (16 pathogenic, 73 neutral) and 72 *BRCA2* variants (5 pathogenic, 67 neutral). In addition to the Classified and the Evaluation Variant Set, we prepared a set of 670 variants of uncertain significance (VUS) from the central registry of GC-HBOC. At the time point of data collection (September 2016), 29,316 families were enrolled in the database. Overall, 899 missense variants were listed, of which 229 were classified as (likely) pathogenic or (likely) benign. Pathogenic missense variants were found in a total of 809 breast cancer and breast and/or ovarian cancer families, 368 of which carried the European founder mutation C61G in *BRCA1* [30].

### Parameter setting

We ran Align-GVD, SIFT, MutationTaster2 and PolyPhen-2 on our data sets with default parameters automatically provided by the Alamut™Visual software. Alamut™provides pre-computed multiple protein alignments serving as the input for Align-GVGD and the SIFT Aligned Sequences tool. We used these orthologous, manually curated alignments as updated on March 17, 2016 (Transcripts: *BRCA1*, NM_007294.3; *BRCA2*, NM_000059.3).

MutationTaster2 was run under specification of Ensembl Transcript IDs as available in Alamut™Visual, i.e., ENST00000357654 (*BRCA1*) and ENST00000380152 (*BRCA2*), and the specification of single base exchanges by position. PolyPhen-2 was called via Batch query using the HumVar-trained model as recommended for the analysis of Mendelian diseases [10]. All analyses were performed on November 27, 2016.

SIFT and MutationTaster2 provide a binary classification into pathogenic and benign variants, i.e., AFFECT PROTEIN FUNCTION or TOLERATED (SIFT) and disease causing or polymorphism (MutationTaster2). In concordance with Moghadasi et al. [4], we defined variants to be classified as pathogenic by Align-GVGD if *C*≥35. In concordance with Leong et al. [13], we defined variants classified as possibly damaging or probably damaging as those found to be deleterious by PolyPhen-2.

### Evaluation strategy

In concordance with Leong et al., Rodrigues et al., and Mueller et al. [13, 15, 31], we evaluated performance on our variant sets based on the following four criteria: (1) sensitivity $\text {SENS}=\frac {\text {TP}}{\text {TP}+\text {FN}}$, (2) specificity $\text {SPEC}=\frac {\text {TN}}{\text {FP}+\text {TN}}$, (3) accuracy $\text {ACC} = \frac {\text {TP} + \text {TN}}{\text {TP}+\text {FP} + \text {TN}+\text {FN}}$ and (4) Matthews correlation coefficient $\text {MCC} = \frac {\text {TP}\ \text {TN} - \text {FP}\ \text {FN}}{\sqrt {(\text {TP}+\text {FP})(\text {TP}+\text {FN})(\text {TN}+\text {FP})(\text {TN}+\text {FN}) }}$, where TP (respectively TN) is the number of true positive (respectively negative) results and FP (respectively FN) is the number of false positive (respectively negative) results.

The MCC is particularly suitable for the evaluation of predictions on imbalanced data [32, 33]. As variant sets for the purpose of evaluation of *in silico* prediction approaches typically show a strong bias towards neutral variants (pathogenic variants are expected to seldom occur), the MCC has been used as a performance measure in a variety of studies on *in silico* prediction approaches [13, 15, 31, 34]. MCC values are defined in a range from -1 (always falsely predicted) to 1 (perfectly predicted) with a value of 0 corresponding to a completely random prediction.

To investigate the performance of combinations of prediction tools, we used the following measures. We defined SENS^*m*,*n*^ and SPEC^*m*,*n*^, with $m,n \in \mathbb {N}, \frac {n}{2}\leq m \leq n$, as the sensitivity and the specificity, respectively, of a combined approach involving *n* prediction tools and classified a variant pathogenic if at least *m* approaches categorized it as pathogenic. We considered all combinations of tools for which $m\geq \frac {n}{2}$ holds, except the case of *m*=2∩*n*=2.

In addition to the evaluation of combined methods on our Evaluation Variant Set we derived a model of the expected performance of combined approaches assuming that the predictions made by individual tools would be absolutely independent. Our theoretical framework is explained in detail in Additional file 2. We are aware that the assumption of independence obviously does not hold true, as all prediction approaches mainly rely on AA conservation in MSAs. However, the assumption of independence between several *in silico* predictions might be a typical misinterpretation by many users, although it represents an unattainable best-case scenario.

## Results

### Performance as stand-alone tools

**Fig. 2:**
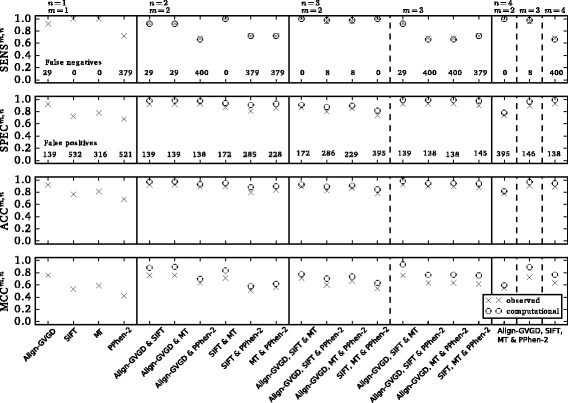
Performance of *in silico* prediction tools as stand-alone methods or in combination. Sensitivity (SENS), specificity (SPEC), accuracy (ACC) and Matthews correlation coefficient (MCC) of stand-alone tools and combinations of prediction tools Align-GVGD, SIFT, MutationTaster2 (MT) and PolyPhen-2 (PPhen-2) as observed and estimated from the sensitivities and specificities of stand-alone methods on the Evaluation Variant Set of 166 missense variants on *BRCA1* and *BRCA2*. Align-GVGD, SIFT and MutationTaster2 reached values for sensitivity > 0.92 as stand-alone tools as well as in combination. The comparatively low sensitivity of PolyPhen-2 as a stand-alone approach is also reflected in the decreased sensitivities of combined approaches involving PolyPhen-2. The specificities of stand-alone tools varied between 0.67 (PolyPhen-2) and 0.92 (Align-GVGD), and the specificities of combined approaches increased with increasing *m*. False negatives (false positives, respectively) denote the number of index patients tested in GC-HBOC as of September 2016 that would receive an erroneous negative (respectively positive) result if the diagnosis were based solely on the corresponding *in silico* approach

**Fig. 3:**
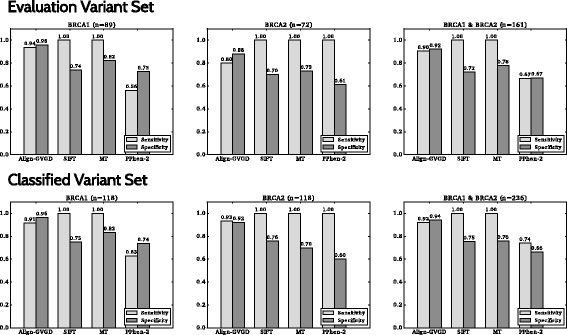
Sensitivities and specificities of the stand-alone prediction tools Align-GVGD, SIFT, MutationTaster2 (MT) and PolyPhen-2 (PPhen-2). The sensitivities and specificities varied between the tools and with values of ≥0.90, Align GVGD performed best for the Evaluation Data Set. Performance for *BRCA1* and *BRCA2* variants was comparable for all tools, except for PolyPhen-2 which showed a lower sensitivity of only 0.56 on *BRCA1* variants compared with 1.0 on *BRCA2* variants and a specificity of 0.67 on *BRCA1* compared with 0.72 on *BRCA2* variants. Comparison between the Classified Variant Set and Evaluation Variant Set revealed only minor differences in sensitivity and specificity for the four tools examined here

Comparing the sensitivity, specificity, accuracy and MCC from our Evaluation Variant Set revealed significant differences in the performance of the prediction tools as stand-alone approaches (Figs. 2 and 3). The sensitivity ranged from 0.67 (PolyPhen-2) to 1.00 (SIFT, MutationTaster2). PolyPhen-2 achieved the lowest sensitivity (0.67), specificity (0.67), accuracy (0.67) and MCC (0.39). A total of 53 variants (32.9%, 7 pathogenic, 46 neutral) from our Evaluation Variant Set were wrongly classified by PolyPhen-2. Furthermore, PolyPhen-2 was unable to correctly predict the effect of the most common pathogenic missense mutation in Germany, C61G in *BRCA1*.

Align-GVGD achieved the highest values of specificity (0.92), accuracy (0.92) and MCC (0.73), but was outperformed regarding its sensitivity (0.90) by SIFT (1.00) and MutationTaster2 (1.00).

By comparing the predictions made by stand-alone tools in our Evaluation Variant Set, we identified 38 out of 140 definitely neutral variants (27.1*%*) that were wrongly classified by at least two of the four prediction programs under investigation. A total of 10 of these 38 variants (7.1% of neutral variants) were misclassified by all four tools. In contrast, we found only one pathogenic missense variant that was wrongly categorized as benign by at least two tools.

### Performance of combined approaches

To investigate the performance of combined approaches in comparison to stand-alone approaches we evaluated the sensitivity, specificity, accuracy and MCC for the Evaluation Data Set. The values observed in our analysis as well as under assumption of the independence of predictions of individual stand-alone tools are visualized in Fig. 2.

Concerning sensitivities, Align-GVGD, SIFT and MutationTaster2 reached values ≥ 0.90 as stand-alone tools as well as in combination. However, the comparatively low sensitivity of PolyPhen-2 as stand-alone approach led to decreased sensitivities of combined approaches involving PolyPhen-2. This result holds true especially in case *m*=*n* (i.e., SENS^2,2^, SENS^3,3^, and SENS^4,4^), as 6 out of 21 pathogenic variants (28.6%) from the Evaluation Variant Set were wrongly classified by PolyPhen-2 while being correctly predicted by Align-GVGD, SIFT and MutationTaster2.

Generally, sensitivities decreased with increasing *m*, namely, SENS^2,3^>SENS^3,3^ and SENS^2,4^>SENS^3,4^>SENS^4,4^. The observed values for the sensitivities of combined approaches were in good agreement with the computed values assuming independence of the predictions of individual approaches.

In contrast to the sensitivities, the specificities of combined approaches increased with increasing *m*, i.e., SPEC^2,3^<SPEC^3,3^ and SPEC^2,4^<SPEC^3,4^<SPEC^4,4^. The contrary effects of the choice of *m* on sensitivity and specificity are quite obvious, as FP can only shrink with increasing *m*, whereas FN may become greater.

Comparing the observed specificities of combined approaches with the corresponding expected values under the assumption of independence of individual predictions, we found a noticeable distinction. Specifically, the expected specificities were consistently greater than the observed ones. This result also held true for the comparison of computed and observed accuracies and MCCs.

### *In silico* identification of benign variants

While the sensitivities achieved by SIFT and MutationTaster2 were 1.00, we observed that the accuracies achieved by all *in silico* approaches under investigation suffer from poor specificities. Due to the relative abundance of benign missense variants these approaches led to a high number of false positive results. Therefore, we examined if *in silico* prediction might be an appropriate approach for the exclusion of pathogenicity, at least. We investigated the suitability of Align-GVGD, SIFT and MutationTaster for determination of benign missense variants as stand-alone tools and in combination. We excluded PolyPhen-2 due to the poor sensitivities we observed. As stand-alone approach, Align-GVGD categorized 131 variants from the Evaluation Variant Set (180 variants from the Classified Variant Set, respectively) as belonging into classes 0, 15 or 25, of which 2 (4) were (likely) pathogenic variants. SIFT classified 101 variants from the Evaluation Variant Set, respectively 141 variants from the Classified Variant Set, as TOLERATED, and MutationTaster2 classified 110 variants from the Evaluation Variant Set, respectively 142 variants from the Classified Variant Set, as polymorphism. All nsSNPs classified as TOLERATED by SIFT or classified as polymorphism by MutationTaster2 were benign variants.

### Possible implications in a diagnostic setting

Examination of the sensitivity, specificity, accuracy and MCC for a set of *BRCA1* and *BRCA2* mutations alone sheds little light on the amounts of patients that would be affected by misleading findings in a clinical setting, e.g., genetic testing in families at risk for breast and ovarian cancer. Because pathogenic as well as benign nsSNPs occur in varying quantities, misleading findings on a single variant may affect different numbers of patients. Hence, we examined how the usage of *in silico* prediction tools would affect the number of false predictions in our cohort of patients fulfilling the inclusion criteria of GC-HBOC for genetic testing. The numbers of patients who would receive a misleading test result with respect to the *in silico* approaches under consideration are reported in Fig. 2.

**Fig. 4:**
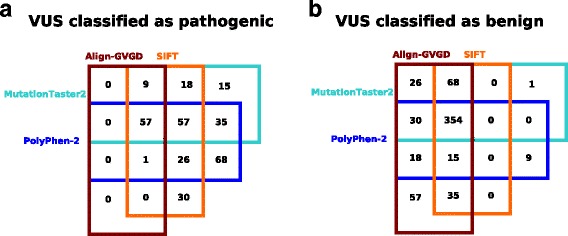
Venn diagrams summarizing the *in silico* prediction on 670 VUS from the GC-HBOC database. Variants classified as pathogenic by at least one program out of Align-GVGD, SIFT, MutationTaster2 and PolyPhen-2 are depicted in **a**), and variants classified as benign are shown in **b**). A total of 354 VUS were consistently classified as benign by all four tools under consideration, and 57 variants were consistently classified as pathogenic. In contrast, 57 variants were classified as benign exclusively by Align-GVGD, whereas 68 (30) were classified as pathogenic exclusively by PolyPhen-2 (SIFT). These inconsistent predictions point toward a noticeable amount of misclassifications by the corresponding tool

We furthermore ran *in silico* prediction on a set of 670 VUS from the GC-HBOC database as of September 2016. 354 variants were consistently classified as benign by all four methods, while 57 variants were consistently classified as pathogenic. However, 259 variants were inconsistently classified by the four tools under investigation (see Fig. 4). If benignity would be assumed for all variants that were consistently classified as benign by SIFT and MutationTaster2, 422 VUS from the GC-HBOC database (62.99%) could be re-classified.

## Discussion

We evaluated the performance of four *in silico* prediction tools for the pathogenicity of missense variants on 161 nsSNPs (Evaluation Variant Set), an enhanced set of 236 nsSNPs (Classified Variant Set), and a set of 670 VUS in *BRCA1* and *BRCA2*. In our study, we focused on a scenario that may typically occur in clinical practice, namely, we used a parameter setting as provided automatically by the Alamut™Visual software. Our findings are in line with a variety of results from similar studies, each uncovering the insufficiency of state-of-the-art prediction tools for medical diagnostics to differing extents. We refer to Additional file 3: Table S2 for a summarizing review. The poor results of PolyPhen-2 when compared with Align-GVGD, SIFT and MutationTaster2 in our study are in agreement with a previous study from Rodrigues et al. [15], as well as with the specificities obtained by Hicks et al., Kerr et al., and Miosge et al. [12, 34, 35]. However, we were not able to confirm the results of Kerr et al. [34] that indicated a poor performance of SIFT on *BRCA1/2* missense variants, especially concerning its specificity.

Obviously, a limitation of our study is the small proportion of pathogenic mutations in our evaluation set, namely, 25 variants (15.1%). However, our approach is justified for the following reasons. First, small numbers of truly pathogenic nsSNPs among the majority of tested missense variants reflect the reality in clinical diagnostics. For example, in the GC-HBOC database as of September 2016, 27.5% of all 229 classified *BRCA1/2* missense variants are ranked as deleterious. Second, evaluation on the Classified Variant Set containing an increased amount of pathogenic nsSNPs (21.1%) revealed results comparable to the evaluation on the original set (see Fig. 3). Third, we refer to the study by Leong et al. [13] which utilized evaluation data with comparatively high amounts of truly pathogenic variants, namely, 92.7% (*KNCQ1*), 91.1% (*KCNH2*), and 87.6% (*SCN5A*). In concordance with our results, Leong and co-workers found sensitivities exceeding the corresponding specificities, with a few exceptions (2 out of 30 experiments with stand-alone tools PolyPhen-2, SNPs&GO, SIFT, PROVEAN and SNAP, data not shown).

For our patients even using the combination of prediction tools resulting in the smallest sum of affected individuals (four tools with *m*=3) would lead to false negative results for 8 patients with pathogenic missense mutations, while false positive results would affect 146 tested individuals with benign results. Our findings are consistent with a publication by Moghadasi and co-workers [4] showing that *in silico* analysis alone is not sufficient to classify 60 VUS in human *BRCA1* and *BRCA2*. In addition, Miosge and co-workers [35] noted that there is a general discordance between affected protein structures and their clinical relevance, as decreased protein functions might be negligible, or compensated for, or require cofactors to result in pathogenicity.

In summary, our findings contribute to the recognition that current state-of-the-art *in silico* prediction tools are inapplicable to determine pathogenicity, especially in a clinical setting. Indeed, the authors of SIFT and PolyPhen-2 explicitly warn against using their tools for this purpose [8, 36]. Combination of several *in silico* approaches did not overcome this drawback. Therefore, determination of pathogenicity should always include additional information like segregation analysis, co-occurrence and functional analyses. However, we addressed for the first time whether *in silico* prediction might be suitable to predict the benignity of missense variants in *BRCA1/2* without the need for further analysis. Our results give reason to assume that *in silico* prediction with SIFT and MutationTaster2 might be an appropriate approach for exclusion of pathogenicity of variants located in highly variable regions, at least. This means, that nsSNPs classified as benign by SIFT or MutationTaster2 are actual benign variants in the overwhelming majority of cases, i.e., false negative predictions are rare. In agreement with our findings, Kerr et al. [34] observed no false negative calls of SIFT in a set of 69 pathogenic *BRCA1/2* nsSNPs. However, confirmation of our hypothesis and its application in clinical diagnostics requires further investigation on larger data sets.

## Conclusions

We conclude that in a routine diagnostic setting the determination of pathogenicity should not be based solely on *in silico* prediction tools as this might result in a large proportion of false positive results and may lead to wrong clinical decisions.

## Additional files

Additional file 1 **Table S1.** List of all variants (*n*=236) from the Classified Variant Set, including reference, functional impact and number of families affected within the cohort of patients from the German Consortium of Hereditary Breast and Ovarian Cancer (as of September 2016). (XLSX 28 kb)

Additional file 2 Supplemental Methods. Explanation of our theoretical model for computation of the expected performance of combined approaches assuming the predictions made by individual tools would be absolutely independent. (PDF 97 kb)

Additional file 3 **Table S2.** The table lists previous studies investigating the performance of Align-GVGD, SIFT, MutationTaster or PolyPhen-2, the characteristics of the data sets utilized, and the observed values. (PDF 79 kb)

## Abbreviations

ACC: Accuracy
ENIGMA: Evidence-based Network for the Interpretation of germline mutant alleles
ExAC: Exome aggregation consortium
FN: False negatives
FP: False positives
GC-HBOC: German consortium for hereditary breast and ovarian cancer
GD: Grantham difference
GV: Grantham variation
HBOC: Hereditary breast and ovarian cancer
InDel: Insertion or deletion
MCC: Matthews correlation coefficient
MSA: Multiple sequence alignments
NGS: Next-generation sequencing
Prior P: Prior probability in the multifactorial probability model for variant classification in *BRCA1/2*
Product of LRs: Product of likelihood ratios
nsSNP: Non-synonymous single nucleotide polymorphism
SENS: Sensitivity
SNP: Single nucleotide polymorphism
SPEC: Specificity
TCGA: The cancer genome atlas
VUS: Variant of uncertain significance
