# Supplementary material for: Performance of in silico prediction tools for the classification of rare BRCA1/2 missense variants in clinical diagnostics
Source: BMC Med Genomics. 2018 Mar 27;11:35. doi: 10.1186/s12920-018-0353-y (PMC5870501; doi:10.1186/s12920-018-0353-y)
Supplement: Supplementary file 2 — Supplemental Methods. Explanation of our theoretical model for computation of the expected performance of combined approaches assuming the predictions made by individual tools would be absolutely independent. (PDF 97 kb) [file 12920_2018_353_MOESM2_ESM.pdf]

Supplement S2: Theoretical framework for determination of expected performance of combined *in silico* approaches

Considering a set of variants S, we denote by $p, 0 \leq p \leq 1$, the fraction of pathogenic variants within S. Hence, S can be non-ambiguously divided into $p|S|$ pathogenic variants and $(1-p)|S|$ non-pathogenic, i.e. benign, variants. We denote the event that a variant originates from the subset of pathogenic (respectively benign) variants by Ω (respectively $\overline{\Omega}$).

Per definition, sensitivity SENS and specificity SPEC can be described by the conditional probabilities $\mathbb{P}(-|\Omega)$ and $\mathbb{P}(+|\overline{\Omega})$, whereby $-$ (respectively $+$) denote that a variant is classified as pathogenic (respectively non-deleterious). Hence, estimators of true positives TP, false positives FP, true negatives TN and false negatives FN are given by

$$\widehat{\text{TP}} = p\ |S|\ \mathbb{P}(-|\Omega) = p\ |S|\ \text{SENS},$$
$$\widehat{\text{FP}} = (1-p)|S|\ \mathbb{P}(-|\overline{\Omega}) = (1-p)|S|(1-\mathbb{P}(+|\overline{\Omega})) = (1-p)|S|(1-\text{SPEC}),$$
$$\widehat{\text{TN}} = (1-p)|S|\ \mathbb{P}(+|\overline{\Omega}) = (1-p)|S|\ \text{SPEC},$$

and

$$\widehat{\text{FN}} = p|S|\ \mathbb{P}(+|\Omega) = p|S|(1-\mathbb{P}(-|\Omega)) = p|S|(1-\text{SENS}).$$

We can therefore derive an estimator of the corresponding accuracy ACC and Matthews correlation coefficient MCC in dependence of SENS and SPEC via

$$\widehat{ACC} = \frac{\widehat{\text{TP}} + \widehat{\text{TN}}}{\widehat{\text{TP}} + \widehat{\text{FP}} + \widehat{\text{TN}} + \widehat{\text{FN}}} = p\text{SENS} + (1-p)\text{SPEC}$$

and

$$\begin{aligned}\widehat{\text{MCC}} &= \frac{\widehat{\text{TP}}\ \widehat{\text{TN}} - \widehat{\text{FP}}\ \widehat{\text{FN}}}{\sqrt{(\widehat{\text{TP}} + \widehat{\text{FP}})(\widehat{\text{TP}} + \widehat{\text{FN}})(\widehat{\text{TN}} + \widehat{\text{FP}})(\widehat{\text{TN}} + \widehat{\text{FN}})}} \\ &= \frac{\sqrt{p(1-p)}(\text{SENS} + \text{SPEC} - 1)}{\sqrt{(p\text{SENS} + (1-p)(1-\text{SPEC}))\left((1-p)\text{SPEC} + p(1-\text{SENS})\right)}}.\end{aligned}$$

It remains the task to describe SENS and SPEC of combined methods in order to derive an estimator of the expected performance of combined approaches under the assumption that the prediction made by individual tools would be absolutely independent. Note that $\text{SENS}^{m,n}$, respectively $\text{SPEC}^{m,n}$,

with $m, n \in \mathbb{N}, \frac{n}{2} \leq m \leq n$ are defined as the sensitivity, respectively specificity, of a combined method involving n approaches and calling a variant as pathogenic in case at least m approaches classify the given variant as pathogenic. We denote particular methods by n_i for $i = 1, \ldots, n$ and denote $-_1$ (respectively $+_i$) as the event that prediction tool n_i classifies a variant as pathogenic (respectively benign). Accordingly, we denote the individual sensitivity and specificity of method n_i as SENS_i and SPEC_i.

We consider all combinations of tools for which $m \geq \frac{n}{2}$ holds, except the case $m = 1 \cap n = 2$. For these combined approaches we defined imputing the independence of individual prediction tools

$$\widehat{\text{SENS}}^{n,n} = \prod_{q=1}^{n} \mathbb{P}(-_q|\Omega) = \prod_{q=1}^{n} \text{SENS}_q \quad \text{for } n \in \{2,3,4\},$$

$$\begin{aligned}\widehat{\text{SENS}}^{n-1,n} &= \widehat{\text{SENS}}^{n,n} + \sum_{q=1}^{n} \left(\mathbb{P}(+_q|\Omega) \prod_{\substack{1 \leq r \leq n \\ r \neq q}} \mathbb{P}(-_r|\Omega) \right) \\ &= \widehat{\text{SENS}}^{n,n} + \sum_{q=1}^{n} \left((1 - \text{SENS}_q) \prod_{\substack{1 \leq r \leq n \\ r \neq q}} \text{SENS}_r \right) \quad \text{for } n \in \{3,4\},\end{aligned}$$

$$\begin{aligned}\widehat{\text{SENS}}^{2,4} &= \widehat{\text{SENS}}^{3,4} + \sum_{q=1}^{4} \left(\mathbb{P}(+_q|\Omega) \sum_{\substack{1 \leq r \leq 4 \\ r \neq q}} \left(\mathbb{P}(+_r|\Omega) \prod_{\substack{1 \leq s \leq 4 \\ s \notin \{q,r\}}} \mathbb{P}(-_s|\Omega) \right) \right) \\ &= \widehat{\text{SENS}}^{3,4} + \sum_{q=1}^{4} \left((1 - \text{SENS}_q) \sum_{\substack{1 \leq r \leq 4 \\ r \neq q}} \left((1 - \text{SENS}_r) \prod_{\substack{1 \leq s \leq 4 \\ s \notin \{q,r\}}} \text{SENS}_s \right) \right),\end{aligned}$$

$$\widehat{\text{SPEC}}^{n,n} = 1 - \prod_{q=1}^{n} \mathbb{P}(-_q|\overline{\Omega}) = 1 - \prod_{q=1}^{n} (1 - \text{SPEC}_q) \quad \text{for } n \in \{2,3,4\},$$

$$\widehat{\mathrm{SPEC}}^{n-1,n} = \widehat{\mathrm{SPEC}}^{n,n} - \sum_{q=1}^{n} \left(\mathbb{P}(+_q|\overline{\Omega}) \prod_{\substack{1\le r\le n \\ r\neq q}} \mathbb{P}(-_r|\overline{\Omega}) \right)$$
$$= \widehat{\mathrm{SPEC}}^{n,n} - \sum_{q=1}^{n} \left(\mathrm{SPEC}_q \prod_{\substack{1\le r\le n \\ r\neq q}} (1-\mathrm{SPEC}_r) \right) \quad \text{for } n \in \{3,4\},$$

and

$$\widehat{\mathrm{SPEC}}^{2,4} = \widehat{\mathrm{SPEC}}^{3,4} - \sum_{q=1}^{4} \left(\mathbb{P}(+_q|\overline{\Omega}) \sum_{\substack{1\le r\le 4 \\ r\neq q}} \left(\mathbb{P}(+_r|\overline{\Omega}) \prod_{\substack{1\le s\le 4 \\ s\notin\{q,r\}}} \mathbb{P}(-_s|\overline{\Omega}) \right) \right)$$
$$= \widehat{\mathrm{SPEC}}^{3,4} - \sum_{q=1}^{4} \left(\mathrm{SPEC}_q \sum_{\substack{1\le r\le 4 \\ r\neq q}} \left(\mathrm{SPEC}_r \prod_{\substack{1\le s\le 4 \\ s\notin\{q,r\}}} (1-\mathrm{SPEC}_s) \right) \right).$$
