# Supplementary material for: Performance of in silico prediction tools for the classification of rare BRCA1/2 missense variants in clinical diagnostics
Source: BMC Med Genomics. 2018 Mar 27;11:35. doi: 10.1186/s12920-018-0353-y (PMC5870501; doi:10.1186/s12920-018-0353-y)
Supplement: Supplementary file 3 — Table S2. The table lists previous studies investigating the performance of Align-GVGD, SIFT, MutationTaster or PolyPhen-2, the characteristics of the data sets utilized, and the observed values. (PDF 79 kb) [file 12920_2018_353_MOESM3_ESM.pdf]

Supplement S3: Evaluation studies for *in silico* prediction tools

	Evaluation data	Align-GVGD			SIFT			MutationTaster2			PolyPhen-2			Remarks
		SENS	SPEC	ACC	SENS	SPEC	ACC	SENS	SPEC	ACC	SENS	SPEC	ACC	
Rodrigoues et al., 2015 [1]	48 variants in *UGT1A1*	0.65	0.67	0.65	0.92	0.80	0.80				0.89	0.40	0.75	with manually curated input alignment of 27 *UGT1A1* orthologs
Mueller et al., 2015 [2]	339 variants in 76 genes				0.56	0.54	0.55				0.48	0.69	0.60	
Luxembourg et al., 2014 [3]	52 variants in *SERPINC1*				0.80	0.67	0.79				0.94	1.00	0.94	
Grimm et al., 2015 [4]	5 data sets with numbers of variants in range from 8850 to 47149				0.62–0.79	0.62–0.80	0.64–0.79	0.74–0.93	0.51 –0.85	0.60–0.88	0.65–0.83	0.62–0.81	0.63–0.82	
Choi et al., 2012 [5]	57646 human missense variants from UniProt				0.85	0.69	0.77*				0.89	0.62	0.76*	*balanced accuracy
Leong et al., 2015 [6]	312 variants in *KCNQ1*, *KCNH2* and *SCN5A*				0.82	0.77	0.81				0.86	0.40	0.81	
Hicks et al., 2011 [7]	33 variants in *BRCA1*, 30 variants in *MSH2*, 60 variants in *MLH1*, and 144 variants in *TP53*	0.00–0.97	0.52–1.00		0.32-0.95	0.18–1.00					0.67–0.97	0.18–1.00		
Miosge et al., 2015 [8]	30 variants in 23 mouse immune genes				1.00	0.58	0.63				1.00	0.42	0.50	nsSNPS were confirmed via *in vivo* tests for loss of function phenotypes
Kerr et al., 2017 [9]	1118 missense variants from the Myriad Genetic Labratories, Inc. database	0.84	0.92	0.91	0.99	0.56	0.60				0.81	0.70	0.71	750 variants were excluded from Align-GVGD evaluation as they were already used for its training

References

[1] Rodrigues, C., Santos-Silva, A., Costa, E., Bronze-da-Rocha, E.: Performance of In Silico Tools for the Evaluation of UGT1a1 Missense Variants. Human Mutation **36**(12), 1215–1225 (2015)

[2] Mueller, S.C., Backes, C., Haas, J., Katus, H.A., Meder, B., Meese, E., Keller, A.: Pathogenicity prediction of non-synonymous single nucleotide variants in dilated cardiomyopathy. Brief Bioinform, 054 (2015)

[3] Luxembourg, B., D'Souza, M., Koerber, S., Seifried, E.: Prediction of the pathogenicity of antithrombin sequence variations by in silico methods. Thromb. Res. **135**(2), 404–409 (2015)

[4] Grimm, D.G., Azencott, C.-A., Aicheler, F., Gieraths, U., MacArthur, D.G., Samocha, K.E., Cooper, D.N., Stenson, P.D., Daly, M.J., Smoller, J.W., Duncan, L.E., Borgwardt, K.M.: The Evaluation of Tools Used to Predict the Impact of Missense Variants Is Hindered by Two Types of Circularity. Human Mutation **36**(5), 513–523 (2015)

[5] Choi, Y., Sims, G.E., Murphy, S., Miller, J.R., Chan, A.P.: Predicting the Functional Effect of Amino Acid Substitutions and Indels. PLOS ONE **7**(10), 46688 (2012)

[6] Leong, I.U., Stuckey, A., Lai, D., Skinner, J.R., Love, D.R.: Assessment of the predictive accuracy of five in silico prediction tools, alone or in combination, and two metaservers to classify long QT syndrome gene mutations. BMC Medical Genetics **16**, 34 (2015)

[7] Hicks, S., Wheeler, D.A., Plon, S.E., Kimmel, M.: Prediction of missense mutation functionality depends on both the algorithm and sequence alignment employed. Hum. Mutat. **32**(6), 661–668 (2011)

[8] Miosge, L.A., Field, M.A., Sontani, Y., Cho, V., Johnson, S., Palkova, A., Balakishnan, B., Liang, R., Zhang, Y., Lyon, S., Beutler, B., Whittle, B., Bertram, E.M., Enders, A., Goodnow, C.C., Andrews, T.D.: Comparison of predicted and actual consequences of missense mutations. Proc Natl Acad Sci U S A **112**(37), 5189–5198 (2015)

[9] Kerr, I.D., Cox, H.C., Moyes, K., Evans, B., Burdett, B.C., Kan, A.v., McElroy, H., Vail, P.J., Brown, K.L., Sumampong, D.B., Monteferrante, N.J., Hardman, K.L., Theisen, A., Mundt, E., Wenstrup, R.J., Eggington, J.M.: Assessment of in silico protein sequence analysis in the clinical classification of variants in cancer risk genes. J Community Genet, 1–9 (2017)
